# Residual Information of Previous Decision Affects Evidence Accumulation in Current Decision

**DOI:** 10.3389/fnbeh.2019.00009

**Published:** 2019-02-05

**Authors:** Farzaneh Olianezhad, Sajjad Zabbah, Maryam Tohidi-Moghaddam, Reza Ebrahimpour

**Affiliations:** ^1^Department of Electrical Engineering, Shahid Rajaee Teacher Training University, Tehran, Iran; ^2^School of Cognitive Sciences, Institute for Research in Fundamental Sciences (IPM), Tehran, Iran; ^3^Department of Computer Engineering, Shahid Rajaee Teacher Training University, Tehran, Iran

**Keywords:** perceptual decision, bias, accuracy, drift-diffusion model, sequential effect

## Abstract

Bias in perceptual decisions can be generally defined as an effect which is controlled by factors other than the decision-relevant information (e.g., perceptual information in a perceptual task, when trials are independent). The literature on decision-making suggests two main hypotheses to account for this kind of bias: internal bias signals are derived from (a) the residual of motor signals generated to report a decision in the past, and (b) the residual of sensory information extracted from the stimulus in the past. Beside these hypotheses, this study suggests that making a decision in the past *per se* may bias the next decision. We demonstrate the validity of this assumption, first, by performing behavioral experiments based on the two-alternative forced-choice (TAFC) discrimination of motion direction paradigms and, then, we modified the pure drift-diffusion model (DDM) based on the accumulation-to-bound mechanism to account for the sequential effect. In both cases, the trace of the previous trial influences the current decision. Results indicate that the probability of being correct in the current decision increases if it is in line with the previously made decision even in the presence of feedback. Moreover, a modified model that keeps the previous decision information in the starting point of evidence accumulation provides a better fit to the behavioral data. Our findings suggest that the accumulated evidence in the decision-making process after crossing the bound in the previous decision can affect the parameters of information accumulation for the current decision in consecutive trials.

## Introduction

Perceptual decisions and their outcomes can be related to each other as a sequence (Hanks et al., 2011; Akaishi et al., 2014; Purcell and Kiani, 2016; Bornstein et al., 2017; Miller et al., 2017). This ability to merge the advance knowledge about choice alternatives with current evidence to make an appropriate decision is a hallmark of higher brain function (Cook and Maunsell, 2002; Roitman and Shadlen, 2002; Gold and Shadlen, 2007; Ratcliff et al., 2007; Churchland et al., 2008; Kiani et al., 2008; Heitz and Schall, 2012). Findings suggest that neural activities in brain areas involved in decision making process contain the history of previous decisions (Boettiger et al., 2007; Serences, 2008; Summerfield and Koechlin, 2008, 2010; Basten et al., 2010; Fleming et al., 2010a,b; Forstmann et al., 2010; Philiastides et al., 2010; Preuschhof et al., 2010; Scheibe et al., 2010; Mulder et al., 2012) (Akaishi et al., 2014) and do not return to the initial value immediately after the time of decision (Cook and Maunsell, 2002; Roitman and Shadlen, 2002; Gold and Shadlen, 2007; Ratcliff et al., 2007; Churchland et al., 2008; Kiani et al., 2008; Heitz and Schall, 2012). Furthermore, there is a preference in humans to repeat their decision, especially when it was made about an ambiguous stimulus (Brehm, 1956; Izuma and Murayama, 2013; Akaishi et al., 2014), on the subsequent trial in the absence of response feedback. This interaction between the history of choices and sensory context, respectively called internal and external signals, is thought to cause the biased decisions about the sensory events (Albright, 2012; Awh et al., 2012; Carnevale et al., 2012; Akaishi et al., 2014).

The mechanism of decision bias as one of the most pervasive biases across many domains of cognitive science, however, remains obscure (Glimcher, 2003; Lauwereyns, 2010; Summerfield and Koechlin, 2010; White and Poldrack, 2014; Hanks and Summerfield, 2017; Kim et al., 2017). Two main hypotheses have been proposed to explain the reasons of this bias, although to date, none of them have been adequately supported. According to the first view, the residual of the sensory information of the previous stimulus causes internal bias signals (Becker, 2008; Pearson and Brascamp, 2008; Sigurdardottir et al., 2008; Albright, 2012; Carnevale et al., 2012). Therefore, a strong sensory signal in the previous trial affects the neural responses (increment in the baseline activity) in the brain sensory areas and the current decision is expected to be made under a larger bias. In the alternative view, the residual of motor response-related signals causes internal bias signals (Gold et al., 2008; Marcos et al., 2013); however, contrary to the first impression, the strength of the sensory signal in the previous trial does not seem to affect the decision-biasing. Akaishi et al. also suggest that, in the absence of response feedback, this bias is a mechanism to update the likelihood of a choice to be made (Akaishi et al., 2014).

Given previous work, we propose the following hypothesis: the residual decision evidence in the previous decision process affects evidence accumulation in the current decision even in the presence of feedback. We tested the validity of this claim using behavioral experiments based on the two-alternative forced-choice (TAFC) discrimination of motion direction and computational modeling. We revealed that, firstly, the probability of being correct in the current decision increases if it is in line with the previous decision, showing a trace from the previous trial on the current one. Secondly, this effect is evident in the presence of the feedback, and is independent of the correctness of the previous decision. Thirdly, excluding the strong stimuli from our analysis amplifies the observed effect. This observation could refer to the repulsive adaptation effect of these strong stimuli (Kohn, 2007). These last two eliminate the effect of the previous stimuli and merely include the decision.

Finally, in order to shed light on the plausible mechanism of the observed effect, we used one successful and the elaborate variant of decision-making models called “drift-diffusion” (Mazurek et al., 2003; Shadlen et al., 2006; Gold and Shadlen, 2007; Voss and Voss, 2007; Kiani et al., 2008; Voss et al., 2013; Tohidi-Moghaddam et al., 2016; Lerche and Voss, 2017; Dully et al., 2018). It has been shown that commitment to a choice is a consequence of a gradual increase in the activity of neurons selective for that specific choice. This gradual increment from a baseline activity is well explained, in this accumulation-to-bound model, by the accumulation of noisy evidence from a starting point which varies depending on the different parameters (Falmagne, 1965, 1968; Remington, 1969; Luce, 1986; Ratcliff et al., 1999; Bogacz et al., 2006; Forstmann et al., 2010; Rorie et al., 2010; Balci and Simen, 2014). In addition, improvement in the activity reaches a stereotyped threshold at decision end (Ratcliff, 1978, 2002; Bogacz et al., 2006; Ratcliff et al., 2016) which corresponds to reaching a specified bound in this model. Our results show that the model that keeps previous decision information in the starting point of accumulation provides a better fit to the behavioral data which support the idea that the activity of decision maker neurons (Gold and Shadlen, 2007) after crossing the bound, in the previous decision, may affect the process of information accumulation of those neurons for the current decision in consecutive trials.

## Materials and Methods

### Participants

In this experiment, six adult participants, three males and three females, with normal or corrected-to-normal vision participated. All the participants, except for two of the middle authors, were unfamiliar with the design of the experiment. They signed informed written consent before attending the study. All experimental protocols were approved by the Iran University of Medical Sciences.

### Visual Stimuli

Random dot motion stimuli are used in a large number of perceptual decision-making studies. These stimuli are movies in which some dots are randomly moving in different direction. In each frame, white dots (2 × 2 pixel, 0.088° per side) were displayed on a black background with a density of 16.7 dots/degree^2^/s (Shadlen and Newsome, 2001; Roitman and Shadlen, 2002). The stimulus contained three interleaved sets of dots displayed on consecutive video frames. Each set was relocated three frames (40 ms) later while a fraction of dots had a coherent continuous motion toward a direction, and the rest of dots were resettled randomly. The stimulus strength was specified by the fraction of dots which moved coherently. Stimulus was presented using a psychophysics toolbox 3.0.12 (Brainard, 1997; Pelli, 1997) for MATLAB R2013a (MATLAB, 2013) on a computer with the operating system of Windows 7 (64-bit), Intel (R), Core (TM) i7, 16 GB internal storage, and NVIDIA Quadro K2000 GPU card.

### Behavioral Task

**Figure 1:**
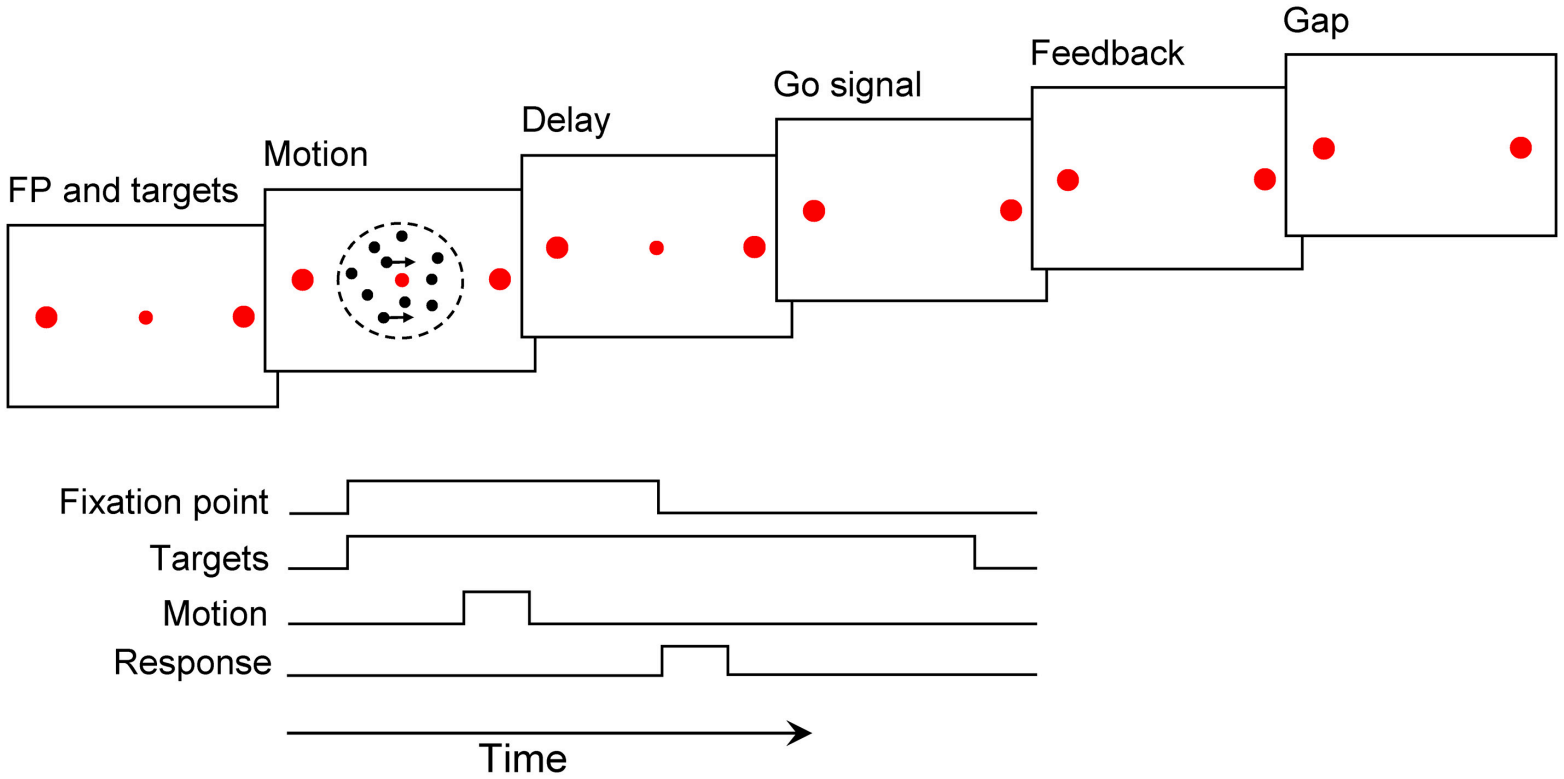
Motion discrimination paradigm. A fixation point (FP) and two targets were presented for 200 ms. After that, the motion stimulus was shown for 120, 400, and 720 ms. The Go signal followed by a 120 ms delay period cued participants to report their decision, within 1 s, by pressing two specific keys. Auditory feedback was played for 100 ms. The following trial began after a gap of 0–1.2 s (see Materials and Methods).

All the experiments were carried out in a semi-dark and sound-proof room. The participants were seated in an adjustable chair at the distance of 57 cm from a cathode ray tube (CRT) display monitor (19 inch, with an 800 × 600 screen resolution, and 75 Hz refresh rate). An adjustable chin-rest had been appropriated to support the participant's chin and forehead. Each trial started with a red fixation point (FP, 0.3° diameter) at the center of the screen and two red choice targets (0.5° diameter) on the right and left side of the fixation point (10° eccentricity). The participants were asked to fix and maintain their gaze on the fixation point throughout the trial. After a 200 ms delay period, the random dots stimulus was displayed within a 5° circular aperture at the center of the screen for 120, 400, and 720 ms. The percentage of coherently moving dots was chosen from these following values: 0, 3.2, 6.4, 12.8, 25.6, and 51.2%. At the end of the stimulus presentation, a 120 ms delay period occurred. After the delay period, the Go signal cued the participants to respond by eliminating the fixation point. The participants were asked to report their decision, about the direction of motion, within 1 second after the Go signal by pressing a left or right key. Distinctive auditory feedback (e.g., beep) was delivered for 100 ms for correct responses, error responses, and missed trials. The type of feedback was chosen randomly for trials with 0% coherence. Trials have been separated by different gap durations: 0, 120, or 1,200 ms. Different gap durations were used to demonstrate their different effects on our results, but there was no significant difference between them, so we have pooled the data of the three gaps in all analysis. The arrangement of the motion direction, motion duration, gap duration, and motion strength varied randomly from trial to trial (Figure 1).

All possible types of trials were randomly interleaved in blocks with 150 trials. The participants were instructed to perform the experiments quickly and accurately to the possible extent. The overall probability of being correct was shown on the screen at the end of each block. Each participant performed one or two sessions (each session had four blocks) per day until 3,600 trials were collected. The participants completed at least one session on each day for six consecutive days. The results were consistent across all participants, but figures have collapsed the data across participants.

### Data Analysis

For the purpose of this study, we focused our analysis on specific pairs of consecutive trials which will be explained along with their reasons in the following. First of all, in order to demonstrate the effect of previous stimulus strengths on the current decision, we picked out the pair of trials in which the first (previous) trial contained two groups of low (0 and 3.2%) and high (12.8 and 51.2%) motion strengths. This categorization is based on the subjects' performance. The performance in 0 and 51.2% is the minimum (50%) and the maximum (100%), correspondingly. The performance in 3.2% (~65%) and 12.8% (~85%) is 15% far from the minimum and the maximum, correspondingly. The second (current) trial consisted of low, middle and nearly high motion strength values (3.2, 6.4, and 12.8%) where the stimulus is not very strong. It also should be noted that in the preliminary analysis, we observed the same results of previous trials which had 25.6%, and 51.2% coherence. Furthermore, we probed previous trials with three different motion durations to illustrate the effect of previous stimulus durations on the current trials with constant motion durations (120 ms); however, no significant difference was found. Accordingly, we have pooled the data of the three motion durations of previous trials in further analysis.

A variety of logistic regression models were used to characterize the effect of different parameters on the probability of correct choice. The following models are fitted by using the generalized linear model (GLM) with binomial error structure. We use *Logit[P]* as a short form of log($\frac{\text{P}}{1-\text{P}}$), and β_*i*_ as fitted coefficients.

**Figure 2:**
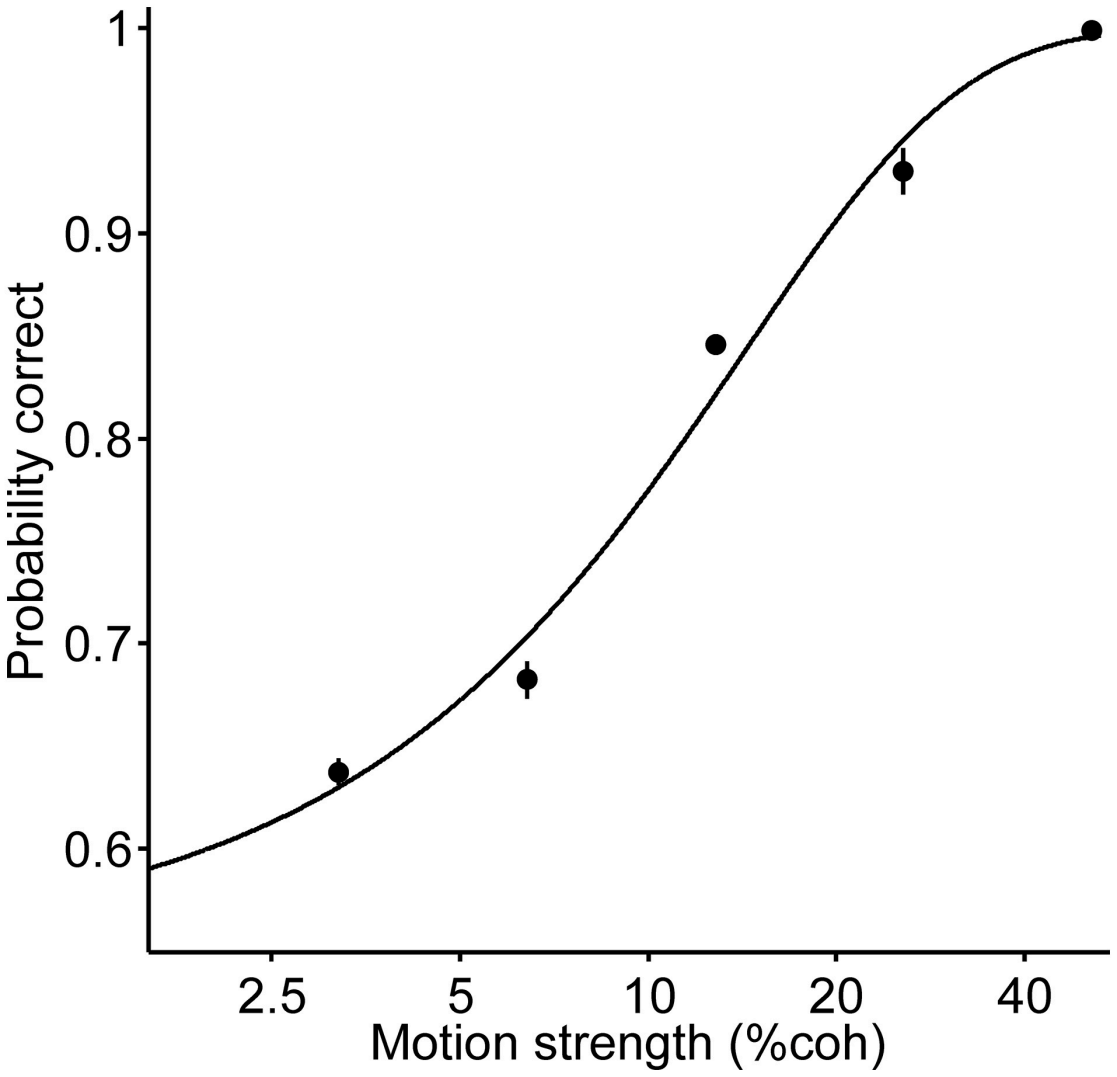
Psychometric function of all the trials; each data point presents the performance of pooled data for all the three durations and two directions. The curve is the fit of a logistic regression to the data (Equation 1). Error bars indicate SE (Standard Error).

**Figure 3:**
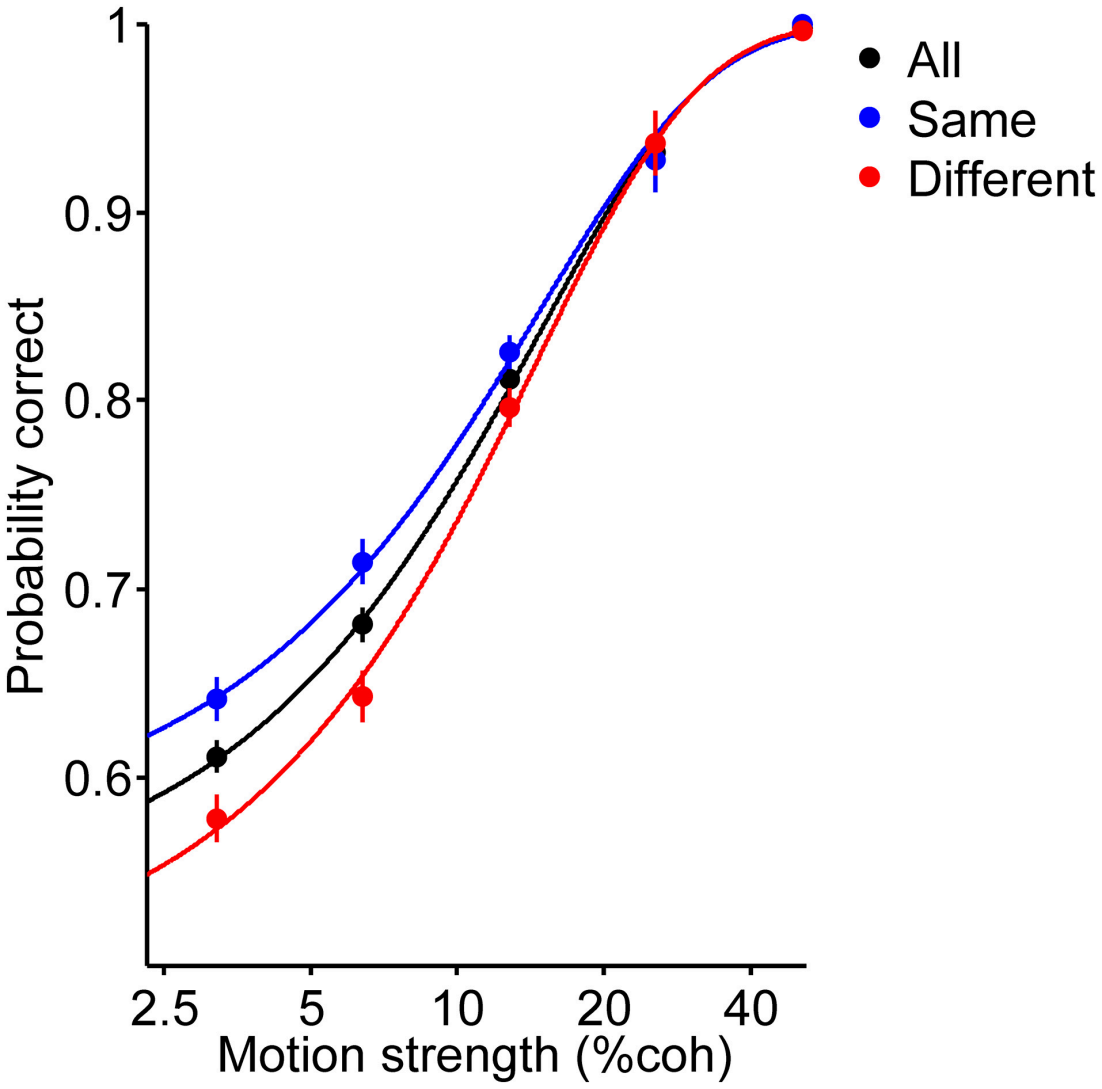
Psychometric function of the current trials. Red and blue data points depict performance of participants for the different and same decision conditions, respectively. Black data points are pooled from these two conditions. Curves are the fit of the logistic regression to the data (Equation 1 for black curve and Equation 2 for red and blue curves). Error bars indicate SE (Standard Error).

The probability of a correct choice is defined by the following (to fit the psychometric function in Figure 2 and for the black curve in Figure 3):

(1) $$\begin{array}{l} {\text{Logit}\left[{\text{P}}_{\text{correct}}\right]=\beta_{0}+\beta_{\text{c}}\text{C}}_{c} \end{array}$$

where *C*_*c*_ is motion strength. To evaluate the effect of the previous decision on the current choice accuracy, we fit the following: (to fit the psychometric function of same and different decision conditions in Figure 3):

(2) $$\begin{array}{l} {\text{Logit}\left[{\text{P}}_{\text{Correct}}\right]=\beta_{0}+\beta_{\text{s}}\text{S}+\beta_{\text{c}}\text{C}}_{c},S=\{\begin{array}{l} 0\text{ different decision}\\ 1\text{ same decision} \end{array} \end{array}$$

where *C*_*c*_ is the motion coherence of the current trials and *S* is an indicator variable for two successive decisions. The null hypothesis is that the current choice accuracy for same and different decision conditions are equal (*H0:* β_*s*_ = *0*).

A modified version of Equation (2) was used to test whether the current choice accuracy was influenced by correctness of the previous trial:

(3) $$\begin{array}{c} {\text{Logit}\left[{\text{P}}_{\text{Correct}}\right]=\beta_{0}+\beta_{\text{s}}\text{S}+\beta_{\text{c}}\text{C}}_{c}+\;{\text{β}}_{\text{e}}\text{E},\\ \text{S}=\left\{ \begin{array}{l} 0\text{ different decision}\\ 1\text{ same decision} \end{array}\right.\text{ E}=\left\{ \begin{array}{l} 0\text{ incorrect previous trial }\\ 1\text{ correct previous trial} \end{array}\right. \end{array}$$

where *C*_*c*_ is the motion coherence of the current trials. *S* and *E* are the indicator variables for two successive decisions and correctness of the previous trials, respectively. The null hypothesis is that the current choice accuracy does not depend on correctness of the previous trial (*H0:* β_*e*_ = *0*).

To examine if the current choice accuracy was affected by two groups of low (0% and 3.2%) and high (12.8% and 51.2%) motion strengths of the previous trial, we altered Equation 2 as follows:

(4) $$\begin{array}{r} {\text{Logit}\left[{\text{P}}_{\text{Correct}}\right]=\beta_{0}+\beta_{\text{s}}\text{S}+\beta_{\text{c}}{\text{C}}_{\text{c}}+\;\beta_{\text{m}}\text{M},}_{\;}^{\;}\\ \text{S}=\{\begin{array}{r} 0\text{ different decision}\\ 1\text{ same decision} \end{array},\\ \text{M}=\left\{ \begin{array}{r} 0\text{ high motion strength in previous trial}\\ 1\text{ low motion strength in previous trial} \end{array}\right. \end{array}$$

where *C*_*c*_ is the motion coherence of the current trials. *S* and *M* are the indicator variables for two successive decisions and motion strengths level of the previous trials, respectively. The null hypothesis is that the current choice accuracy does not depend on motion strengths level of the previous trial (*H0:* β_*m*_ = *0*).

To assess the impact of the motion strength of the previous trial on the current choice accuracy we used the following regression:

(5) $$\begin{array}{l} \text{Logit}\left[{\text{P}}_{\text{Correct}}\right]=\beta_{0}+\beta_{\text{p}}{\text{C}}_{\text{p}}+\beta_{\text{c}}{\text{C}}_{\text{c}} \end{array}$$

where *C*_*p*_ and *C*_*c*_ are the motion coherence of the previous and current trials, respectively. The null hypothesis is that previous stimulus strength has no significant effect on current choice accuracy (*H0:* β_*p*_ = *0*).

We compared the accuracy in the same decision condition (the blue curve in Figure 3) to the accuracy in different decision condition (the red curve in Figure 3) using logistic regression, as follows:

(6) $$\begin{array}{l} \text{Logit}\left[{\text{P}}_{\text{s}}\right]=\beta +\text{Logit}\left[{\text{P}}_{\text{d}}\right] \end{array}$$

where *P*_*s*_ and *P*_*d*_ are the probability correct in the same decision condition and different decision condition, respectively. The null hypothesis is that the accuracies in both conditions are equal (*H0:* β = *0*).

All statistical analyses were performed in R version 3.3.1 (The R Foundation for Statistical Computing, www.R-project.org). The statistical analyses outcomes are presented in the RESULTS section.

### Modeling

In order to investigate the mechanism of the last decision impact on the current decision, we used the drift-diffusion model (DDM) (Ratcliff, 1978; Ratcliff and McKoon, 2008) as implemented by Voss et al. in a computationally efficient, flexible and user-friendly program called fast-dm (Voss and Voss, 2007). Fast-dm estimated DDM's parameters using the partial differential equation (PDE) method through fast computations to calculate the cumulative distribution function (CDF) and the Chi-Square statistic (Voss et al., 2013; Lerche and Voss, 2017).

Undoubtedly, the diffusion model is a well-established model in the perceptual decision literature (Gold and Shadlen, 2007; Voss et al., 2013). This model consistently explains both neural and behavioral responses, and its different parameters can explain the process of commitment to a choice in the brain based on an accumulation-to-bound mechanism (Mazurek et al., 2003; Shadlen et al., 2006; Gold and Shadlen, 2007; Voss and Voss, 2007; Kiani et al., 2008). In the pure drift-diffusion model (DDM), momentary sensory evidence in favor of one of the choices starts to accumulate from a baseline point (*z*). Just after the integrated evidence over time (guided by drift rate *v*) hits a criterion level or bound (*a*), the decision process is terminated (Ratcliff, 1978; Ratcliff and McKoon, 2008; Ratcliff et al., 2016). Seven parameters that exist in the full DDM are divided into three categories: (1) the decision process parameters (decision bound *a*, mean baseline point *z*, and mean drift rate *v*), (2) the non-decision process parameter (non-decision time *t*_*ND*_ ), (3) the variability across-trial parameters (variability in stimulus quality η, variability in baseline point *sz*, and variability in non-decision time *st*_*ND*_) (Ratcliff, 1978; Ratcliff and Tuerlinckx, 2002; Ratcliff and McKoon, 2008). According to the proposed hypothesis in the present research, the previous decision can influence the current decision process in three possible ways: (a) the previous decision affects the rate of accumulated evidence (i.e., the drift rate, ν) (Ashby, 1983; Ratcliff, 1985; Diederich and Busemeyer, 2006; Bornstein et al., 2017), (b) it changes the mean baseline point of evidence accumulation (*z*) (Edwards, 1965; Laming, 1968; Link and Heath, 1975; Ratcliff, 1985; Voss et al., 2004; Bogacz et al., 2006; Diederich and Busemeyer, 2006; Wagenmakers et al., 2008; Bornstein et al., 2017), or (c) it shifts the decision threshold (*a*) (Ratcliff and Rouder, 1998; Ratcliff and Smith, 2004; Bogacz et al., 2006; Simen et al., 2006; Goldfarb et al., 2012). The diffusion model along with a model comparison method (Smith and Spiegelhalter, 1980; Kass and Wasserman, 1995; Liddle, 2007) will be used to disentangle these three scenarios (Falmagne, 1965, 1968; Remington, 1969; Ratcliff, 1985; Luce, 1986; Ratcliff et al., 1999; Ratcliff and Smith, 2004).

## Results

### Behavior

Six human participants reported the perceived direction of motion in trials with 120, 400 and 720 ms duration (Figure 1). The psychometric function for the participants is shown in Figure 2. The psychometric function of current trials separated in the three conditions is plotted in Figure 3. The first condition or the so called same decision condition, blue data points, shows the performance of current trials in which the participants have taken a decision similar to the previous trial. In the second condition or different decision condition, red data points, the participants' decisions in current trials are different from those in the previous trials. The third condition, black data points, is the performance of all current trials, independent of the decision in previous trials. Considering the black data points as a reference, an upward and a downward shift is obvious in the psychometric function of the same and different decision conditions, respectively. Generally, it can be said that upward and downward shifts which occurred in Figure 3 are independent of the current stimuli with low and middle motion strength values (Equation 6, β = 0.27 ± 0.03, *p* = 3.2 × 10^−16^, positive β indicates accuracies in the same decision condition are higher than the accuracies in the different decision condition). This shift is not evident in the strong stimuli of the current trials (25.6 and 51.2%) because, in the salient stimuli which are not ambiguous, the decision is more dependent on the sensory information (Akaishi et al., 2014). Thus, detecting any kinds of bias is much more difficult in such stimuli. As a result, we focused our analysis on ambiguous stimuli in the current trials.

This difference between psychometric functions of the same and different decision conditions implies that not only does the probability of being correct in a decision depend on the stimulus strength, but also on the previous decision (Equation 2, β_*s*_ = 0.25 ± 0.09, *p* = 5.8 × 10^−8^). Indeed, the probability of being correct in the current trial will increase (decrease) if the reported direction in the current decision and the chosen direction in the previous trial are alike (different).

One may conclude that this difference in performance is the effect of stimulus adaptation since the previous decision is itself correlated to the previous stimulus. Interestingly, the reported effect of the previous decision seems to be in contrast with the repulsive effect of adaptation. Taking the repulsive effect into account, we expect higher sensitivity for the perception of leftward (rightward) motion when it comes after a rightward (leftward) motion. As a result, the probability of being correct should be higher in the different decision condition compared to the same one. In what follows, we tried to elaborate on these two probable contradictory effects through further analysis.

**Figure 4:**
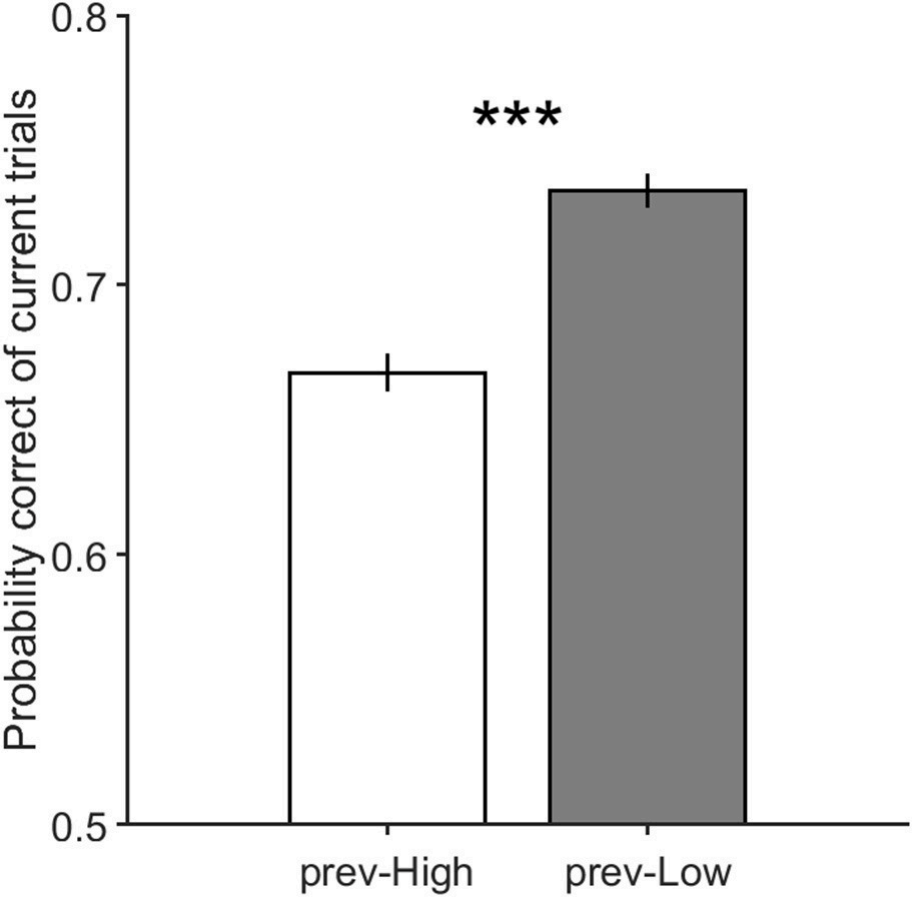
The performance of the current trials which includes motion strengths of 3.2, 6.4, and 12.8% when their previous trials have high motion strengths of 12.8, 25.6, and 51.2% in prev-High, and have low motion strengths of 0 and 3.2% in prev-Low. Error bars indicate SE (Standard Error). Wilcoxon rank-sum test is used to test the significance of the difference, ***p < 1E−3.

It is worth noting that in case there is an adaptation effect in our paradigm, it should be stronger when the stimulus of the previous trial has high motion coherence. In order to investigate whether there is any adaptation effect in our data, we separated trials with high and low motion strength in their previous trials and compared the performance of the current trials in these two conditions. As shown in Figure 4, the accuracy of trials which preceded by high coherence stimuli is significantly lower than the accuracy of those preceded by low coherence stimuli. This result supports the presence of the adaptation and its strong effect in trials preceded by high coherence stimuli. Therefore, to untwist the same/different decisions effect from the sensory adaptation effect, we separated high and low coherence stimuli from the preceding analysis, and calculated how the same and different decision conditions differ in performance.

**Figure 5:**
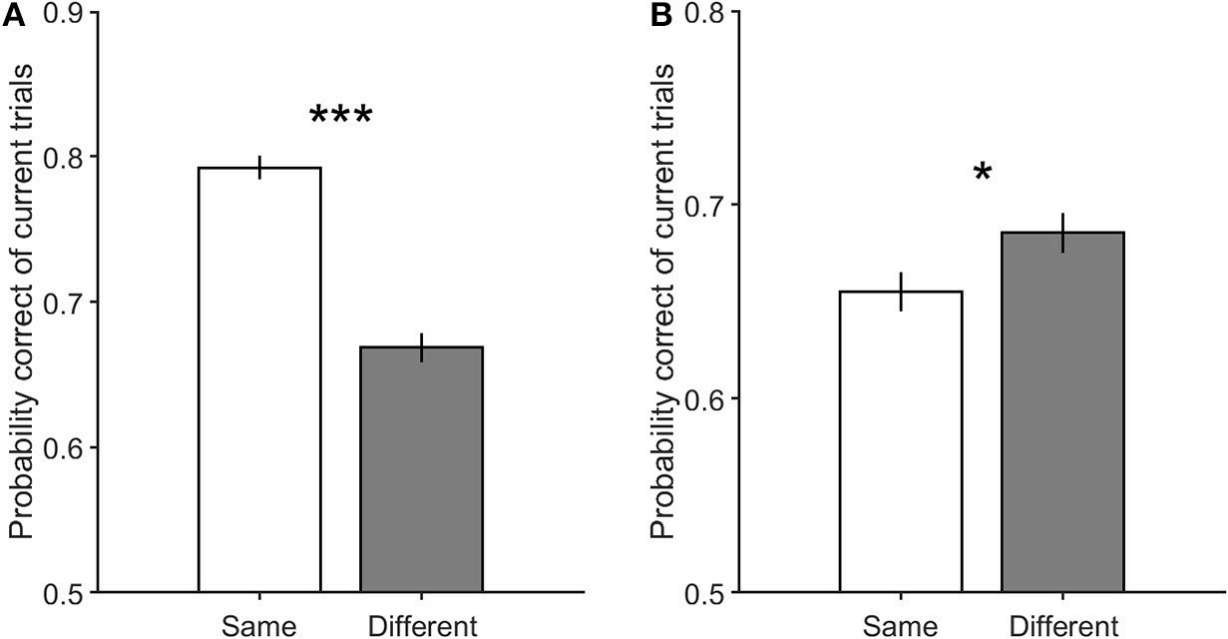
The performance of the current trials with motion strengths of 3.2, 6.4, and 12.8%. **(A)** shows performance in the current trials when previous trials have low motion strengths of 0 and 3.2%. **(B)** illustrates performance in the current trials when previous trials have high motion strengths of 12.8 and 51.2%. Error bars indicate SE (Standard Error). Wilcoxon rank-sum test is used to test the significance of the differences, *p < 0.05, ***p < 1E−3.

**Figure 6:**
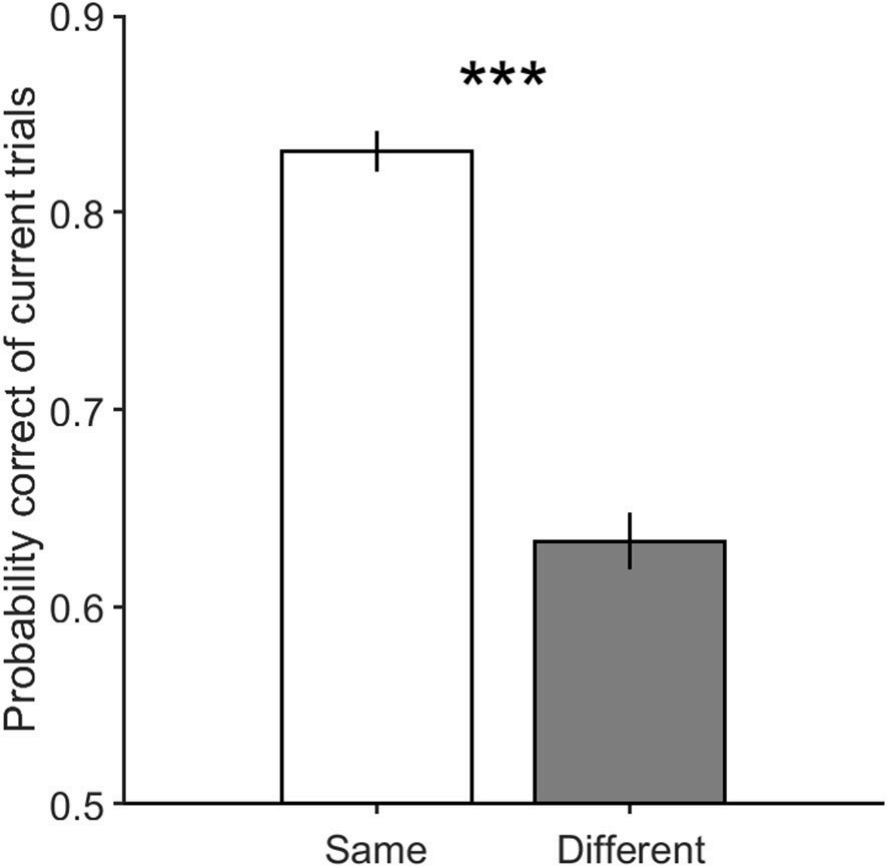
The performance of the current trials which includes motion strengths of 3.2, 6.4, and 12.8% when their previous trials are 0%. Error bars indicate SE (Standard Error). Wilcoxon rank-sum test is used to test the significance of the difference, ***p < 1E−3.

Figure 5 illustrates the performance in current trials which includes motion strengths of 3.2, 6.4, and 12.8%, when previous trials have low motion strengths of 0 and 3.2% (Figure 5A, Equation 2, β_*s*_ = 0.65 ± 0.13, *p* = 4.3 × 10^−22^) and high motion strengths of 12.8 and 51.2% (Figure 5B, Equation 2, β_*s*_ = −0.14 ± 0.13, *p* = 0.03). As shown in this figure, the participants are significantly more likely to choose a correct decision in the different decision condition when the coherence of the previous trial is high, which is consistent with the repulsive adaptation effect. Whereas, the Figure 5A shows that a correct decision is more probable in the same decision condition when stimulus coherence in the previous trial is low (Equation 4, β_*m*_ = 0.28 ± 0.09, *p* = 1.5 × 10^−9^). Moreover, this observed effect is significant even when previous trials have 0% coherence in which all dots move randomly, and minimizes the adaptation in any specific direction. However, as illustrated in Figure 6, the probability of being correct is greater in the same decision condition than in the different decision condition, even when there is lack of coherent motion (motion strengths of 0%) in the previous stimulus (Equation 2, β_*s*_ = 0.98 ± 0.19, *p* = 2.2 × 10^−23^). Therefore, decreasing the effect of stimulus adaptation by excluding previous trials with high coherence stimulus seems to strengthen the effect of the previous decision presented in Figure 3.

**Figure 7:**
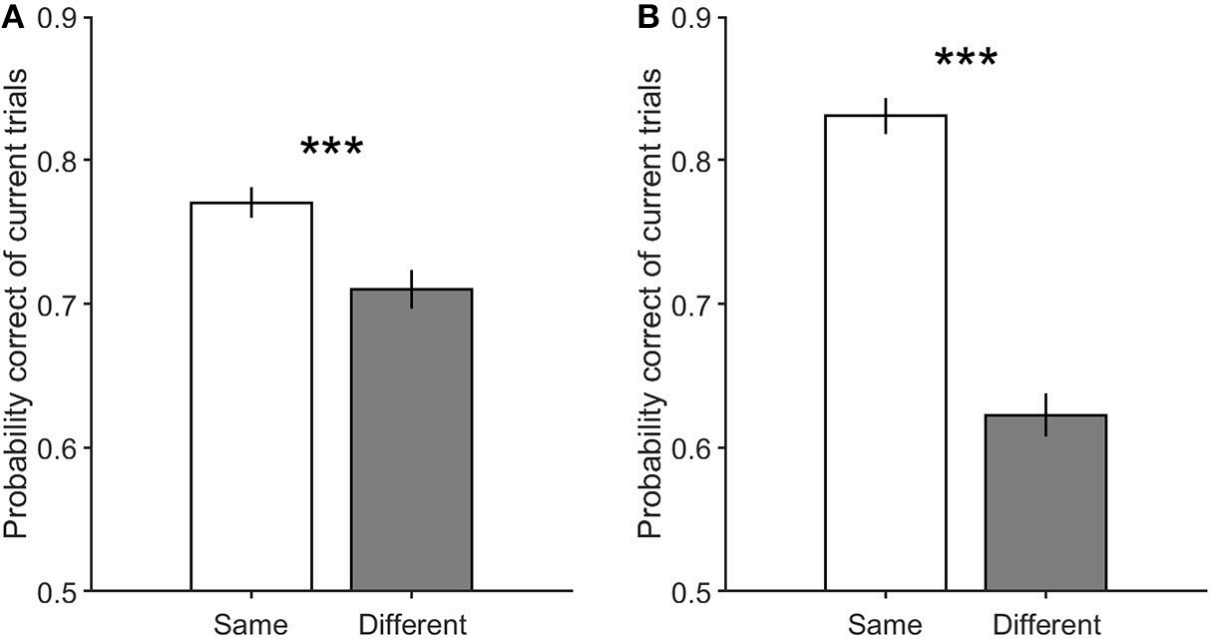
The performance of the current trials which include motion strengths of 3.2, 6.4, and 12.8%. **(A)** is the performance of the current trials when their previous trials are correct with low motion strengths (0 and 3.2%). **(B)** is the performance of the current trials when their previous trials are incorrect with low motion strengths (0 and 3.2%). Error bars indicate SE (Standard Error). Wilcoxon rank-sum test is used to test the significance of the differences, ***p < 1E−3.

Another salient point that may contribute to the current decision accuracy difference between the same and different decision conditions is the previous trial's feedback. As stated before, the feedback signal is different in the correct and incorrect trials, and may result in the observed effect. Here in Figure 7, by separating correct and incorrect previous trials in both the same and different decision conditions, we attempted to eliminate the influence of the feedback. As illustrated in this figure, the correctness of the previous decision does not remove the effect explained above (Equation 3, β_*e*_ = −0.07 ± 0.1, *p* = 0.19). In other words, similar decision trials are significantly more probable to be correct than different decision trials, regardless of the previous decision to be correct (Figure 7A, Equation 2, β_*s*_ = 0.37 ± 0.17, *p* = 3.3 × 10^−5^) or incorrect (Figure 7B, Equation 2, β_*s*_ = 1.06 ± 0.21, *p* = 3.6 × 10^−22^).

### Model Fits

As indicated previously, to investigate the underlying mechanism of the previous decision's effect on the probability of being correct in the current choice, we used the drift-diffusion model (DDM). Dependence of the model parameters on the previous decision gave us the chance to examine the effect of the previous decision on each parameter. To do so, besides the pure DDM, we ran three modified versions of it, and fit these four models to the behavioral data derived from experimental study to provide further intuition into the nature of the observed effect.

The first model (model_p_) is the pure DDM in which the only dependent variable, drift-rate (*v*), depends on the current motion strength. In the second model (model_v_), as a modified DDM, *v* depends on both current motion strength and previous decision (same and different decision conditions). The third one (model_z_) is a drift-diffusion model in which the starting point of evidence accumulation (*z*) is dependent on the previous decision, and *v* depends on the current motion strength. The fourth DDM (model_a_) modified by the dependence of the decision bound (*a*) on the previous decision, as well as *v* is dependent on the current motion strength.

Fitted parameters of each model are listed in Tables 1–4 (mean±SE across participants). For each participant's details, see Tables S1–S4. Here, *s* and *d* indices respectively stand for the same and different decision conditions. As Table 2 shows, based on the dependence of the drift-rate on both current motion strength and previous decision, there are six different drift-rates for three current stimulus coherences (3.2%, 6.4%, and 12.8%) and two conditions (same and different). Regarding the parameters of the third model in Table 3, there are two different starting points for the same and different decision conditions. As presented in Table 4, model_a_ has two different decision threshold related to two different decision conditions.

**Table 1:** Fitted parameters (mean ± SE) of the pure DDM (model_p_).

*z*	0.555 ± 0.015
*a*	0.681 ± 0.062
*v_3.2_*	0.321 ± 0.081
*v_6.4_*	0.780 ± 0.113
*v_12.8_*	1.855 ± 0.155
*t_*ND*_*	0.178 ± 0.008
*st_*ND*_*	0.116 ± 0.012

**Table 2:** Fitted parameters *(mean* ± *SE)* of the second DDM (model_v_).

*z*	0.551 ± 0.013
*a*	0.690 ± 0.610
*v_3.2_*s*_*	0.488 ± 0.104
*v_6.4_*s*_*	1.023 ± 0.136
*v_12.8_*s*_*	1.907 ± 0.195
*v_3.2_*d*_*	0.201 ± 0.058
*v_6.4_*d*_*	0.525 ± 0.100
*v_12.8_*d*_*	1.748 ± 0.147
*t_*ND*_*	0.176 ± 0.008
*st_*ND*_*	0.120 ± 0.011

**Table 3:** Fitted parameters *(mean* ± *SE)* of the third DDM (model_z_).

*z__*s*_*	0.566 ± 0.014
*z__*d*_*	0.540 ± 0.013
*a*	0.688 ± 0.061
*v_3.2_*	0.333 ± 0.070
*v_6.4_*	0.755 ± 0.105
*v_12.8_*	1.804 ± 0.135
*t_*ND*_*	0.176 ± 0.008
*st_*ND*_*	0.121 ± 0.011

**Table 4:** Fitted parameters *(mean* ± *SE)* of the fourth DDM (model_a_).

*z*	0.553 ± 0.013
*a__*s*_*	0.685 ± 0.064
*a__*d*_*	0.688 ± 0.059
*v_3.2_*	0.324 ± 0.070
*v_6.4_*	0.758 ± 0.104
*v_12.8_*	1.806 ± 0.131
*t_*ND*_*	0.176 ± 0.008
*st_*ND*_*	0.120 ± 0.011

As we expected from behavioral results, which indicated the current decision had higher accuracy when the selected direction was similar to the reported one in the previous trial compared to when they were different, the drift-rate and starting point obtained bigger values in the same decision condition in comparison to the different decision condition provided that they are dependent on the previous decision. On the contrary, the decision threshold in the same decision condition is smaller than its value in the different decision condition.

Models have been compared using the Bayes Information Criterion (BIC) (Smith and Spiegelhalter, 1980; Kass and Wasserman, 1995; Liddle, 2007) for the different model fits which are exposed in Table 5 (mean ± sd across participants). As shown in this table, the overall quality of the fits was good (*R*^2^ > 0.83). For details of subjective scores, see Tables S5–S9.

**Table 5:** Model performance comparison via BIC and *R*^2^ metrics (mean ± sd across participants).

**Model**	**Total parameters**	***R*^**2**^**	**BIC**
Model_p_	7	0.836 ± 0.111	−26.726 ± 6.113
Model_v_	10	0.951 ± 0.026	−29.902 ± 5.570
Model_z_	8	0.965 ± 0.016	−34.858 ± 3.267
Model_a_	8	0.843 ± 0.024	−25.406 ± 1.374

Furthermore, BIC values were compared using a Student's *t*-test. Accordingly, the modified DDM with the dependent starting point, model_z_, received the smallest BIC compared to the model_p_ (*p* = 5.6 × 10^−3^) and model_a_ (*p* = 3.2 × 10^−4^). Except for the first participant, all other five participants yielded the lower BIC for model_z_ than model_v_ (see Tables S6, S7). However, the comparison of overall BIC scores showed marginal significant lower BIC for model_z_ compared to model_v_ (*p* = 0.051). After excluding the first participant, the model_z_ led to a significant lower BIC value than model_v_ (*p* = 0.03). Eventually, we chose the model_z_ with the best explanation for how the current choice accuracy is influenced by previous decision.

**Figure 8:**
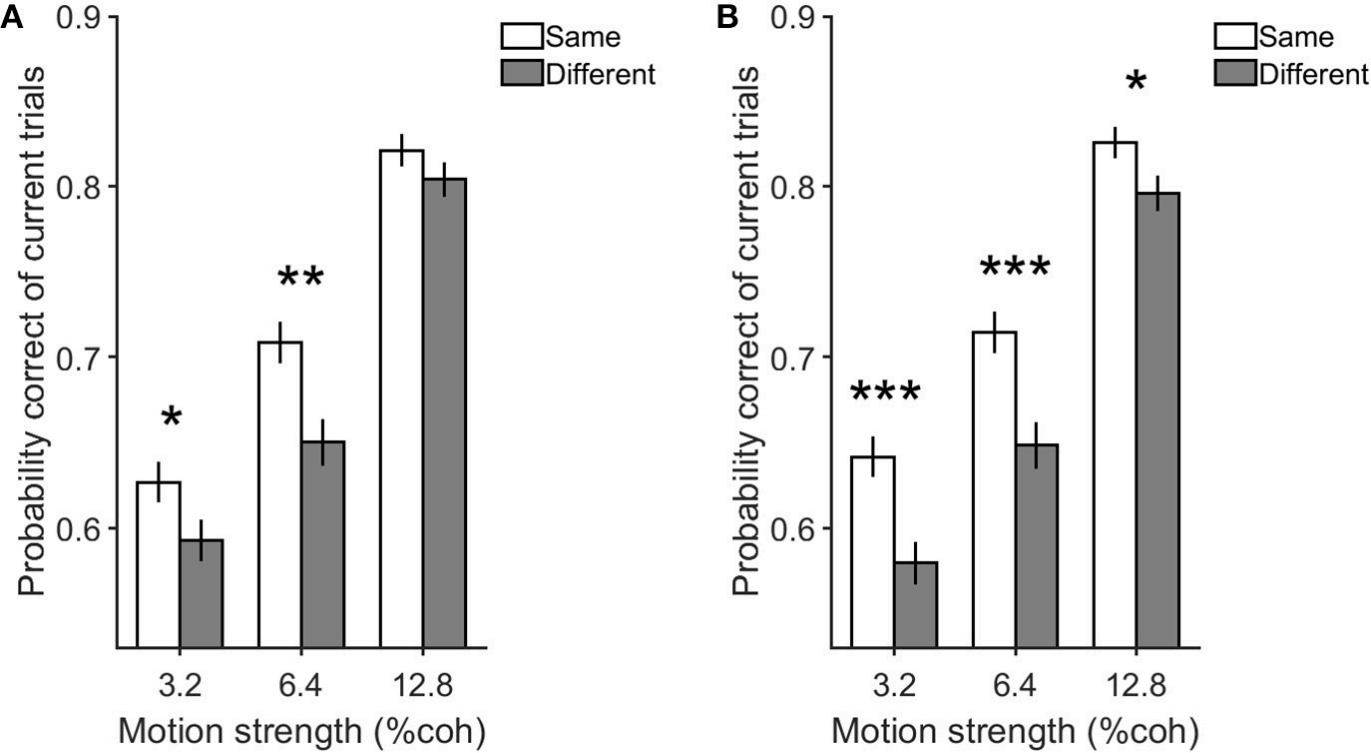
Simulation and behavioral data for the previous decision effect on the current one in the same and different decision conditions. Either panel indicates the performance of the current trials with motion strengths of 3.2, 6.4, and 12.8% when previous trials have low motion strengths of 0 and 3.2%. **(A)** is the simulation data by model_z_ and **(B)** is the experimental data. Error bars indicate SE (Standard Error). Wilcoxon rank-sum test is used to test the significance of the differences, *p < 0.05, **p < 1E−2, ***p < 1E−3.

In that case, we have provided more insight into the model_z_ through simulation. The model_z_ parameters were applied to obtain model performance individually for each of the conditions while the same order of the stimulus in the behavioral experiment was used as an input to this model. As illustrated in Figure 8, consistent with the behavioral results, the same decision condition in the model resulted in the greater accuracy of current trials compared to the different decision condition (*p* = 2 × 10^−4^).

In the latest step, we investigated the difference of the dependent parameter in different conditions for the winner model (model_z_). As stated before, starting point gained higher value in the same decision condition (*z*__*s*_) compared to the different decision condition (*z*__*d*_), and it's consistent across all participants except participant 3 (for participants' details, see Tables S3). Focusing on the data of this participant, it seems that participant 3 is influenced by the sensory adaptation effect even in 3.2%. As shown in Figure S1, the accuracy of the current trials is higher in the same decision condition compared to the different decision condition only when previous trials have 0% motion strengths in which all dots had random movements. The effect of decision bias will be twisted with the effect of the sensory adaptation through pooling the data of these two panels, and that is why the starting point in the same decision condition is not significantly higher than its value in the different decision condition. The significance of the differences between *z*__*s*_ and *z*__*d*_ was tested by the nonparametric bootstrap method (Efron and Tibshirani, 1994; Hinkley, 1998). These differences were quite significant (*p* < 1.7 × 10^−6^) for every five participants.

## Discussion

Our results showed, in sequential perceptual decisions, the probability of being correct in the current choice increases if it is similar to the previous one and conversely decreases when they are different. Although many studies suggested that sequential effects (Falmagne, 1965, 1968; Remington, 1969; Gold et al., 2008; Goldfarb et al., 2012) on decision processes are due to the motor response bias or sensory bias (Gold et al., 2008; Pearson and Brascamp, 2008; Albright, 2012; Carnevale et al., 2012; Marcos et al., 2013), Akaishi et al. showed that this decision history effect cannot be defined through these biases, as well as it can be explained by an autonomous learning rule to estimate the likelihood of a choice to be made (Akaishi et al., 2014). Besides, considering the fact that the firing rate of decision maker neurons cannot meet their baseline activity immediately after the decision (Cook and Maunsell, 2002; Roitman and Shadlen, 2002; Gold and Shadlen, 2007; Ratcliff et al., 2007; Churchland et al., 2008; Kiani et al., 2008; Heitz and Schall, 2012), we hypothesized that the bound crossing in the previous decision provides information which affects the state of decision variable in the subsequent decision.

To verify this assertion, we presented the results of a behavioral study of decision-making using 2AFC paradigm based on randomly moving dots with fixed duration and short interval time, focusing on sequences of two trials. To study the potentially plausible mechanisms accounted for the variations in the probability of correct due to the sequential effect (Falmagne, 1965, 1968; Remington, 1969; Ratcliff, 1985; Luce, 1986; Ratcliff et al., 1999; Ratcliff and Smith, 2004), we extended the pure DDM (Ratcliff, 1978, 2002; Ratcliff and Tuerlinckx, 2002; Bogacz et al., 2006). In the extended versions of DDM, different free parameters of the model were depended on the previous decision. We also indicated the model with dependent baseline has the best explanation for the observed changes in participants' performance for the same and different decision conditions. The results supported our hypothesis that the state of decision variable at the beginning of the information accumulation is being affected by the decision in the previous trial.

**Figure 9:**
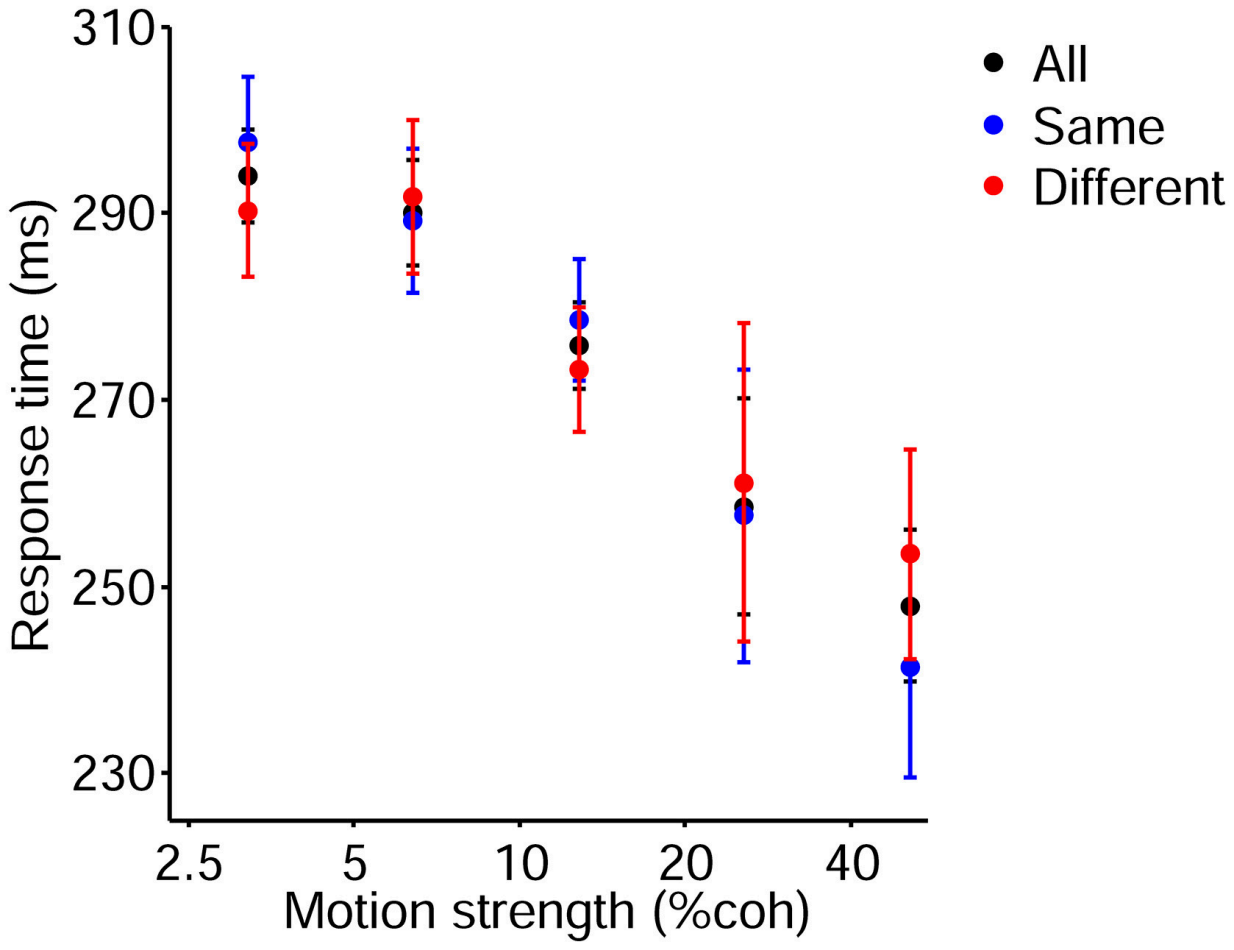
Psychometric function of the current trials which includes motion strengths of 3.2, 6.4, 12.8, 25.6, and 51.2%. Red and blue data points depict response time of participants for the different and same decision conditions, respectively. Black data points are pooled from these two conditions. Error bars indicate SE (Standard Error).

It should be noted that, to avoid increasing the time between consecutive decisions, we utilized fixed duration task which had fixed period for each part of a trial and limited Go signal. Indeed, we tried to prevent lengthening the time between previous bound crossing and start of the current decision process for the sake of preserving the previous decision effect. Nevertheless, we recorded the response time (time elapsed from Go signal onset to a hand key-press) besides the choice accuracy in our experiment. As shown in Figure 9, response times decreased with increasing strength of motions (Link, 1992; Roitman and Shadlen, 2002; Ratcliff and Smith, 2004), and were used as the input data of the models in addition to the current choice accuracy, current stimulus strength, and previous decision although there was no significant difference in them in different decision conditions due to fixed duration task.

With respect to all results elaborated on this study, in a comparative approach, we investigated the sequential effect on the probability of being correct in the current decision in contrast to what Akaishi et al. (2014) indicated the impact of the previous decision on the choice repetition probability. In addition, they did not use feedback in their experiments and declared that the mechanism which is associated with making an incorrect choice rather than recognition of an error is responsible for the decision bias (Akaishi et al., 2014), whereas we claimed that the decision, independent of the correctness and having positive or negative feedback, affects the probability of being correct in the next decision (as shown in Figure 7). Consequently, to support this statement we did another analysis by separating correct and incorrect previous trials with 0% motion strength in both the same and different conditions. Actually, we duplicated Figure 7 only for 0% coherent motion of previous trials (See the Figure S2). In these trials, all dots had random movements which prevented the sensory bias in any particular direction and feedback was given randomly to the participants, independently of whether they pressed the left or the right key. So, the participants received positive feedback on 50% of the trials (Figure S2A) and negative feedback on the other 50% of the trials (Figure S2B). As demonstrated in Figure S2, similar decision trials are significantly more probable to be correct than different decision trials regardless of the previously received feedback.

Since there is a clear feedback after each trial one may conclude that the main finding is due the win-stay lose-switch strategy where subjects tend to repeat their decision after a receiving a correct feedback and tend to switch their decision after receiving a wrong feedback. However, as shown in Figure 7 same decisions have higher performance than different decisions for both correct and wrong previous trials. Thus, the effect is not due to the win-stay lose-switch strategy.

Our results can rule out the effect of sensory bias in three ways: (1) We discussed that there is a sensory bias in our results but that is in the opposite direction of our main effect. We showed that for the strong previous stimulus the effect is diminished. Moreover, the main effect is strongest when the previous stimulus is 0% which is not expected due to a sensory bias. (2) In the modeling part, we examined a model with different drift rates as a model for sensory bias but it cannot better explain the data than the other model. (3) We stated that the stimulus duration does not change the main effect which is in contrast to our expectation of the sensory bias.

Furthermore, we designed a control experiment to dissociate the effect of the previous decision from the motor response bias. In this experiment, the relationship between the decision and motor response is altered pseudorandomly across trials. Accordingly, one of the participants performed a version of the main task in which the right and leftward arrows were used above and below the fixation point as the choice targets (see the Figure S3). The arrangement of these two arrows changed pseudorandomly across trials. The participant was asked to report her decision about the direction of motion by pressing the upper and lower buttons, which arranged vertically and correspond to the position of the arrows, with the right middle and index fingers, respectively. Three thousand and six hundred trials (in 4 blocks × 6 sessions) were collected. As shown in Figure S4, the probability of being correct is significantly larger in the same decision condition compared to the different decision condition, even when the participant made a different motor response to report her perceived motion direction. In consequence, the motor response bias cannot account for the previous decisions' effect.

Although Equation 5, as a simple regression, illustrated that the strength of the stimulus in the previous trial does not affect the performance of current trial (Equation 5, β_*p*_ = −0.003 ± 0.002, *p* = 0.017), separating high and low motion strengths in previous trials demonstrated that the probability correct of current trial is influenced by the previous decision. As a result, it suggests that the sequential effect should be considered in the perceptual decision-making tasks. For instance, the time between consecutive trials should be adjusted properly to keep down the previous decision effect. Two main contributions of the observed sequential effect are emphasized here. First, it originated from the previous decision which was made about the weak stimulus. The analysis of previous trials which consisted of 0% motion strength (Figure S2) showed that not only this sequential effect cannot be defined by the sensory bias, but also it stems from the previous decision which affects the parameters of evidence accumulation in the current decision. Second, the feedback did not play a key role in the effect of previous decisions, since the changes of current decision parameters were independent of the participants' awareness of their correct and incorrect previous decisions.

## Author Contributions

FO has contributed to the conception and study design, data collection, data analysis and interpretation, statistical analysis, modeling and drafting. MT-M has contributed to the study design, data collection, interpretation of data and drafting. SZ has contributed to the study design, drafting, modeling and interpretation of data. RE has contributed to the design of the work and interpretation of data. All authors have approved this final version of the manuscript to be published.

### Conflict of Interest Statement

The authors declare that the research was conducted in the absence of any commercial or financial relationships that could be construed as a potential conflict of interest.

## Abbreviations

ms: millisecond
sd: standard deviation
BIC: Bayes Information Criterion
CDF: Cumulative Distribution Function
CRT: Cathode Ray Tube
DDM: Drift-Diffusion Model
GLM: Generalized Linear Model
PDE: Partial Differential Equation
R^2^: R squared
SE: Standard Error
TAFC: Two-Alternative Forced-Choice.
