# Supplementary material for: Residual Information of Previous Decision Affects Evidence Accumulation in Current Decision
Source: Front Behav Neurosci. 2019 Feb 5;13:9. doi: 10.3389/fnbeh.2019.00009 (PMC6371064; doi:10.3389/fnbeh.2019.00009)
Supplement: Supplementary file 1 [file Data_Sheet_1.pdf]

SUPPLEMENTARY MATERIAL

Figure S1. The performance of the participant 3 on the current trials which includes motion strengths of 3.2%, 6.4%, and 12.8%. **(A)** shows performance in the current trials when previous trials have low motion strengths of 0%. **(B)** shows performance in the current trials when previous trials have low motion strengths of 3.2%. The figure shows that the accuracy of the current trials is higher in the same decision condition compared to the different decision condition only when previous trials have 0% motion strengths in which all dots had random movements. Error bars indicate SE (Standard Error). Wilcoxon rank-sum test is used to test the significance of the differences, $^{*}p<0.05$.

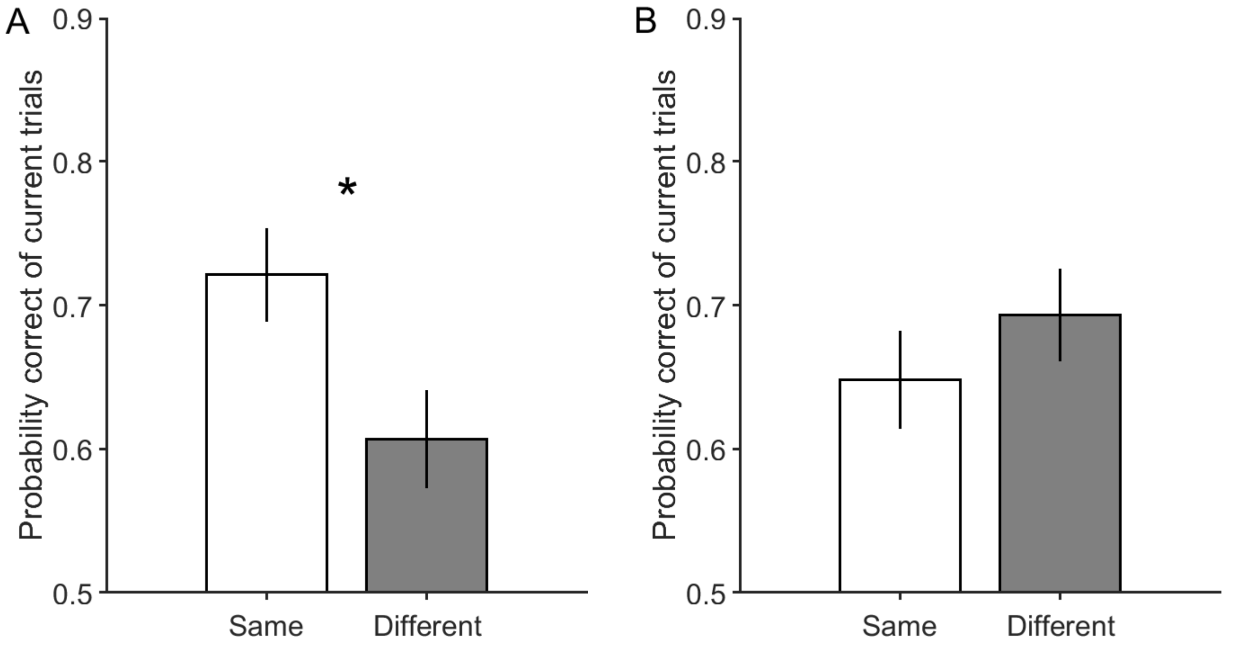

Figure S2. The performance of the current trials which includes motion strengths of 3.2%, 6.4%, and 12.8%. **(A)** is the performance of the current trials when participants have received positive feedback in their previous trials with 0% motion strengths. **(B)** is the performance of the current trials when participants have received negative feedback in their previous trials with 0% motion strengths. The figure shows that the accuracy of the current trials is higher in the same decision condition compared to the different decision condition for both positive and negative feedback of previous trials in which all dots had random movements. Error bars indicate SE (Standard Error). Wilcoxon rank-sum test is used to test the significance of the differences, ****p<1E-3.*

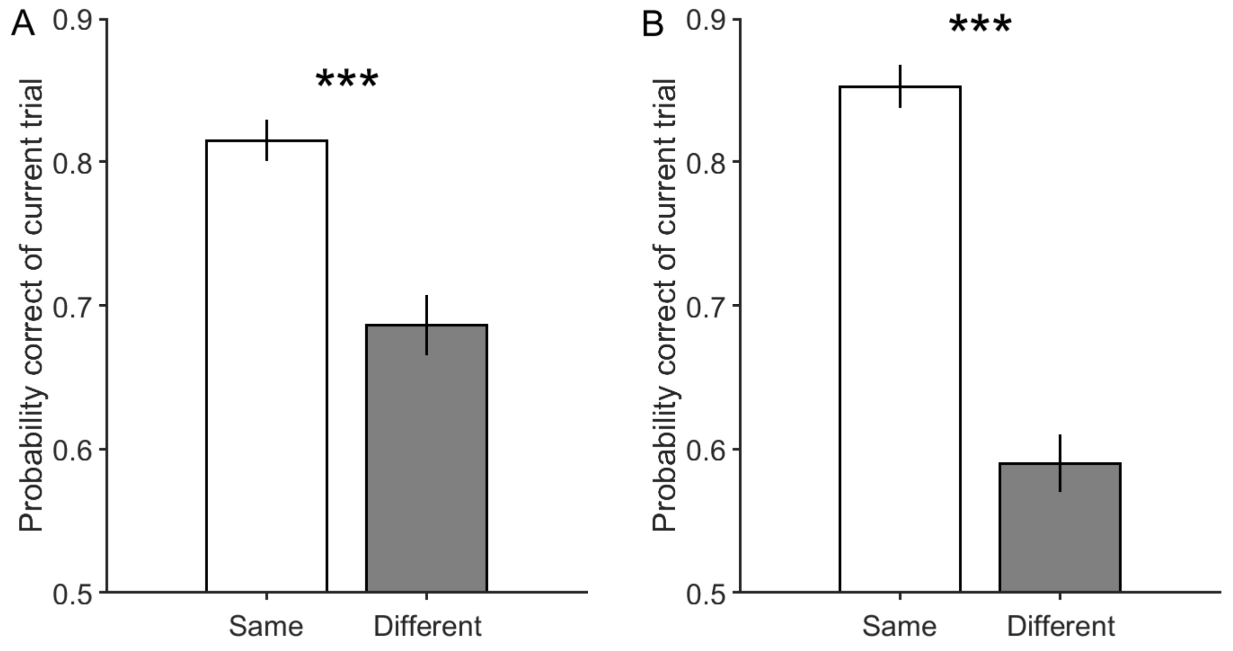

Figure S3. Motion discrimination paradigm with a random response map. A fixation point was presented for 200 ms. After that, the motion stimulus was shown for 120 ms, 400 ms and 720 ms. The right and leftward pointing arrows as the choice targets appeared in a 120 ms delay period. Elimination of the fixation point in the Go signal cued participant to report her decision, within 1 second, by pressing two specific keys which arranged vertically. Auditory feedback was played for 100 ms. The following trial began after a gap of 0-1.2s.

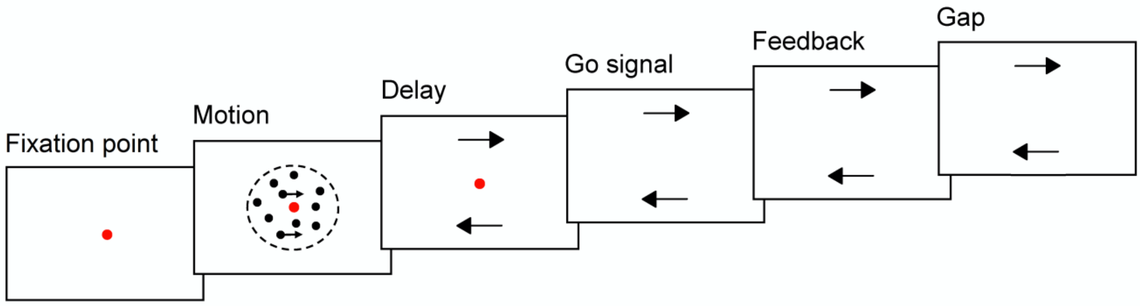

Figure S4. The performance of a single participant on the current trials with motion strengths of 3.2%, 6.4%, and 12.8% when their previous trials have low motion strengths of 0% and 3.2% in the control experiment which dissociate the effect of the previous decision from the motor response bias. Error bars indicate SE (Standard Error). Wilcoxon rank-sum test is used to test the significance of the differences, **p<1E-2.

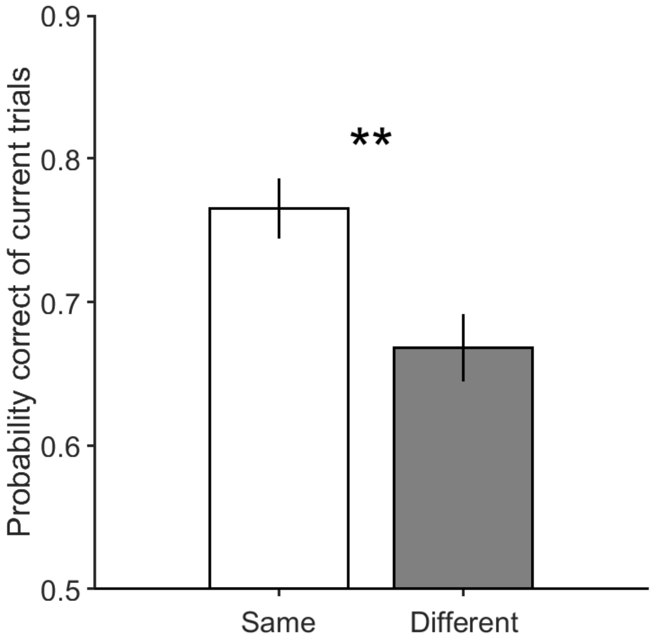

Table S1. Fitted parameters of the pure DDM (model$_p$) for each participant.

Participant/Param	z	a	$v_{3.2}$	$v_{6.4}$	$v_{12.8}$	t_{ND}	st_{ND}
Participant1	0.5757	0.7747	0.2314	0.6983	1.3883	0.1668	0.0946
Participant2	0.6044	0.5359	0.0372	0.2906	1.7864	0.1784	0.1041
Participant3	0.5392	0.5962	0.5474	0.8147	1.8796	0.1914	0.1061
Participant4	0.5093	0.9441	0.4968	0.7984	1.5067	0.2021	0.0866
Participant5	0.5238	0.6178	0.4163	1.0545	2.2366	0.1481	0.1519
Participant6	0.5754	0.6197	0.1944	1.0252	2.3346	0.1815	0.1529

Table S2. Fitted parameters of a modified DDM with dependent drift rate for each participant (model$_v$).

Participant/Param	z	a	$v_{3.2\_s}$	$v_{3.2\_d}$	$v_{6.4\_s}$	$v_{6.4\_d}$	$v_{12.8\_s}$	$v_{12.8\_d}$	t_{ND}	st_{ND}
Participant1	0.5714	0.7794	0.4546	0.0758	1.0585	0.3735	1.5416	1.2578	0.1661	0.0982
Participant2	0.5978	0.5359	0.0307	0.1508	0.4784	0.1541	1.7485	1.8046	0.1774	0.1052
Participant3	0.5336	0.6090	0.6759	0.4599	0.9468	0.5567	1.6235	2.2559	0.1890	0.1046
Participant4	0.5123	0.9484	0.6824	0.2563	0.9557	0.5835	1.5348	1.4291	0.2013	0.1000
Participant5	0.5275	0.6376	0.6800	0.0847	1.4836	0.5980	2.3109	1.7955	0.1427	0.1538
Participant6	0.5607	0.6271	0.4061	0.1756	1.2132	0.8829	2.6838	1.9459	0.1777	0.1579

Table S3. Fitted parameters of a modified DDM with dependent starting point for each participant (model$_z$).

Participant/Param	$z_{\_s}$	$z_{\_d}$	a	$v_{3.2}$	$v_{6.4}$	$v_{12.8}$	t_{ND}	st_{ND}
Participant1	0.5977	0.5526	0.7760	0.2431	0.6958	1.3884	0.1673	0.1000
Participant2	0.6087	0.5869	0.5363	0.0902	0.3156	1.7734	0.1774	0.1048
Participant3	0.5336	0.5432	0.6088	0.5571	0.7199	1.8557	0.1890	0.1047
Participant4	0.5277	0.5000	0.9459	0.4833	0.7681	1.4828	0.2022	0.1023
Participant5	0.5509	0.5046	0.6311	0.3713	1.0218	2.0842	0.1439	0.1569
Participant6	0.5794	0.5513	0.6274	0.2556	1.0087	2.2369	0.1777	0.1580

Table S4. Fitted parameters of a modified DDM with dependent threshold for each participant (model$_a$).

Participant/Param	z	$a_{\_s}$	$a_{\_d}$	$v_{3.2}$	$v_{6.4}$	$v_{12.8}$	t_{ND}	st_{ND}
Participant1	0.5719	0.7788	0.7744	0.2410	0.7159	1.4022	0.1663	0.0981
Participant2	0.6000	0.5417	0.5263	0.0740	0.3096	1.7693	0.1777	0.1059
Participant3	0.5399	0.5830	0.6296	0.5257	0.7371	1.8807	0.1902	0.1062
Participant4	0.5125	0.9567	0.9321	0.4923	0.7810	1.4910	0.2018	0.0979
Participant5	0.5275	0.6408	0.6217	0.3714	1.0135	2.0592	0.1437	0.1535
Participant6	0.5679	0.6061	0.6423	0.2391	0.9901	2.2335	0.1788	0.1567

Table S5. BIC and R^2 metrics of pure DDM ($model_p$) for each participant.

Participant/Criteria	R^2	BIC
Participant1	0.7599	-23.7996
Participant2	0.9667	-36.0490
Participant3	0.8746	-27.9836
Participant4	0.7809	-22.7395
Participant5	0.6809	-19.0968
Participant6	0.9451	-30.6871

Table S6. BIC and R^2 metrics of $model_v$ for each participant.

Participant/Criteria	R^2	BIC
Participant1	0.9878	-38.0690
Participant2	0.9805	-35.6845
Participant3	0.9341	-28.2624
Participant4	0.9397	-26.9006
Participant5	0.9377	-25.1812
Participant6	0.9261	-25.3169

Table S7. BIC and R^2 metrics of $model_z$ for each participant.

Participant/Criteria	R^2	BIC
Participant1	0.9724	-36.7816
Participant2	0.9736	-37.4458
Participant3	0.9517	-33.7043
Participant4	0.9685	-34.3841
Participant5	0.9411	-29.1008
Participant6	0.9830	-37.7341

Table S8. BIC and R^2 metrics of $model_a$ for each participant.

Participant/Criteria	R^2	BIC
Participant1	0.8256	-25.7166
Participant2	0.8323	-26.3561
Participant3	0.8496	-26.8934
Participant4	0.8139	-23.7183
Participant5	0.8549	-23.6904
Participant6	0.8814	-26.0633

Table S9. BIC Differences (ΔBIC) between all four models and for each participant.

Model/Participant	Participant1	Participant2	Participant3	Participant4	Participant5	Participant6
$Model_p$	14.269	1.397	5.721	11.645	10.004	7.047
$Model_v$	0	1.761	5.442	7.484	3.920	12.417
$Model_z$	1.287	0	0	0	0	0
$Model_a$	12.352	11.090	6.811	10.666	5.410	11.671
